# Bleomycin, vincristine, cisplatin/bleomycin, etoposide, cisplatin chemotherapy: an alternating, dose intense regimen producing promising results in untreated patients with intermediate or poor prognosis malignant germ-cell tumours

**DOI:** 10.1038/sj.bjc.6601528

**Published:** 2004-02-03

**Authors:** D A Anthoney, M J McKean, J T Roberts, A W Hutcheon, J Graham, W Jones, J Paul, S B Kaye

**Affiliations:** 1 Department of Medical Oncology, Beatson Oncology Centre, Glasgow, UK; 2 Northern Centre for Cancer Treatment, Newcastle upon Tyne, UK; 3 Department of Medical Oncology, Aberdeen Royal Infirmary, Aberdeen, UK; 4 Department of Medical and Clinical Oncology, Cookridge Hospital, Leeds, W Yorkshire LS16 6QB, UK

**Keywords:** malignant germ cell tumour, poor prognosis, chemotherapy, dose intense

## Abstract

Patients with poor and intermediate prognosis metastatic germ-cell tumours (MGCTs) are at a significant risk of relapse after standard platinum-based chemotherapy. Novel treatment regimens are required to improve survival. Dose intense, alternating combinations of drugs with known activity in germ-cell tumours represents one approach. In all, 43 patients with IGCCCG intermediate/poor prognosis MGCT were treated with a dose intense regimen alternating bleomycin, vincristine, cisplatin (BOP) with bleomycin, etoposide, cisplatin (BEP) to a maximum of three cycles. Data were collected on the maintenance of dose intensity, toxicity, response, progression-free (PFS) and overall survival (OS). The complete response rate was 58%; a further 7% of patients being rendered disease free by resection of viable residual tumour. With a median follow-up of more than 4 years in surviving patients, 3-year OS and PFS rates of 81% (95% CI: 66–91%) and 72% (95% CI: 56–83%) are seen, respectively. Bleomycin, vincristine, cisplatin (BOP)/bleomycin, etoposide, cisplatin (BEP) was well tolerated, with 86% of patients completing all planned courses. Toxicity was predominantly haematological with common toxicity criteria grade III neutropenia in 90% of patients. Cisplatin neuropathy and bleomycin-induced pulmonary toxicity represented the most significant nonhaematological toxicity. Bleomycin, vincristine, cisplatin (BOP)/bleomycin, etoposide, cisplatin (BEP) represents a practicable, well-tolerated, dose intense chemotherapy regimen with significant activity in intermediate and poor prognosis MGCT.

The introduction of effective cisplatin-based chemotherapy regimens since the 1970s, has converted metastatic germ-cell tumours (MGCTs), with a very poor prognosis, into the paradigm of a curable cancer. Despite the development of strategies that successfully treat the majority of patients with MGMT, there remains a small but significant group of patients destined to die of their disease. Considerable effort has thus been directed at refining the treatment of individual patients depending on their risk of relapse or treatment failure, predicted from prognostic factors present at the time of diagnosis. The development of a prognostic factor-based staging system by the International Germ Cell Cancer Collaborative Group (IGCCCG, 1997) has provided a standardised set of prognostic indicators that identify intermediate prognosis and poor prognosis groups.

Although the salvage of relapsed MGCT is increasingly successful (De Bono *et al*, 2000), strategies to improve the survival from intermediate or poor prognosis MGCT have focused predominantly on first-line chemotherapy regimens. Standard therapy for nonmetastatic, nonseminomatous germ-cell tumours at high risk of relapse, and for good prognosis MGCT has, in general, consisted of a combination of cisplatin, etoposide and bleomycin (BEP). Approaches to improve on this have been to increase cisplatin dose (Ozols *et al*, 1983); add or substitute novel chemotherapeutic agents (de Wit *et al*, 1999); alternate treatment regimens between combinations of active agents (Bower *et al*, 1997; Germa *et al*, 1999); increase the dose intensity by shortening intervals between treatments (Horwich *et al*, 1989; Horwich *et al*, 1997); use myeloablative doses of active agents in combination with bone marrow or peripheral stem-cell rescue (Motzer *et al*, 1997; Decatris *et al*, 2000); or combinations of these methods. To date, none of these have proven superior to standard BEP therapy in prospective randomised trials. However, we believe that an intensive, alternating schedule represents one of the most promising approaches.

The aim of this study was, therefore, to determine the toxicity and effectiveness, in terms of the response and survival of alternating BOP/BEP, a novel dose intense combination chemotherapy regimen, based on individual components with which there was already considerable experience.

## PATIENTS AND METHODS

### Patients

Patients aged ⩽65 years with IGCCCG intermediate or poor prognosis MGCT were eligible for this study. Histological diagnosis of primary testicular or extragonadal germ-cell cancer was required, although diagnosis on the strength of elevated serum tumour markers (*α*FP, *β*hCG, LDH) alone was allowable if tissue for histology was unobtainable. Minimum characteristics for intermediate or poor prognosis disease were defined as: testis/retroperitoneal primary and AFP ⩾1000 ng ml^−1^; or HCG ⩾5000 IU l^−1^; or LDH⩾1.5 × upper limit of normal. Prior chemotherapy or radiotherapy was not allowed and disease-specific surgery was restricted to orchidectomy or biopsy of tumour. Patients were required to have adequate renal function (calculated GFR ⩾40 ml min^−1^) prior to trial entry.

### Ethics review

The protocol was approved by the local research ethics committee of each participating institution. Written informed consent was obtained from each patient.

### Treatment

**Figure 1:**
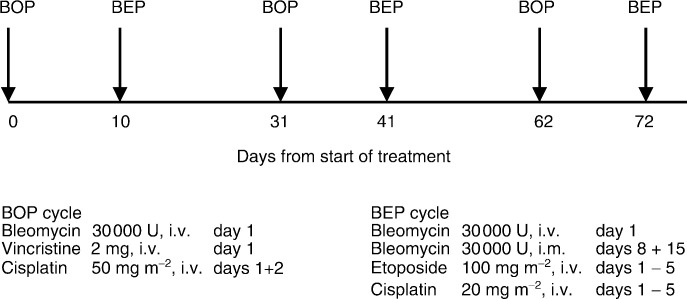
BOP/BEP chemotherapy regimen.

The BOP/BEP regimen is shown in Figure 1

. An initial course of BOP was followed on day 10 by a course of standard 5-day BEP (de Wit *et al*, 2001). The BOP cycle was repeated again on days 31 and 62, with BEP on days 41 and 72.

In the BOP regimen, cisplatin at 50 mg m^−2^ was administered over 4 h on each of 2 days with a standard pre- and postchemotherapy hydration regimen, and after ensuring an adequate urine output (>100 ml h^−1^). For BEP, cisplatin at 20 mg m^−2^ was given daily for 5 days by the same method. Intravenous bleomycin (30 000 U) was administered as a 12 h infusion in 500 ml of normal saline.

### Treatment modifications

A total white blood cell count (WBC) <1.5 × 10^9^ l^−1^ or platelets <50 × 10^9^ l^−1^ on the first day of any treatment cycle resulted in a treatment delay. Blood counts were repeated every 3 days until these thresholds were exceeded and treatment was given. A delay of more than 2 weeks resulted in withdrawal from the study.

Cisplatin dose was reduced by 25% for a WBC of 1.5–2.0 × 10^9^ l^−1^ and/or platelets of 50–75 × 10^9^ l^−1^ on the day of treatment. At blood counts higher than these cisplatin was given at full dose.

A combination of WBC 1.5–2.0 × 10^9^ l^−1^ and platelets 76–100 × 10^9^ l^−1^ or WBC >2.1 × 10^9^ l^−1^ and platelets 50–75 × 10^9^ l^−1^ on the day of treatment reduced etoposide dose by 50%. For WBC 1.5–2.0 × 10^9^ l^−1^ and platelets >100 × 10^9^ l^−1^ or WBC >2.1 × 10^9^ l^−1^ and platelets 76–1000 × 10^9^ l^−1^, the reduction of etoposide dose was 25%.

Prophylactic use of white cell colony-stimulating factors was not permitted, although they could be used at the discretion of the treating doctor in the event of prolonged or complicated neutropenia.

Creatinine clearance was calculated (Cockcroft and Gault formula) at the start of each treatment. Cisplatin and bleomycin were withheld if the calculated clearance was <40 ml min^−1^. If renal function recovered to >40 ml min^−1^, cisplatin was restarted at 75% and bleomycin at 100%. Severe skin toxicity (⩾common toxicity criteria (CTC) grade 2) or signs of lung toxicity were indications for stopping bleomycin.

### Assessment of response and toxicity

A total of three complete cycles of BOP and BEP was planned for each patient. A complete response was classified as normalisation of tumour markers with no clinical or radiological evidence of residual disease at the completion of treatment. Clinical or radiological evidence of residual tumour, despite normal markers, was an indication for explorative surgery with resection and histological examination of the residual masses. The absence of viable tumour cells, or the presence of mature teratoma only, in the resected tumour was also classified as a complete response. An incomplete response to treatment was defined as the presence of residual viable cancer in resected material postchemotherapy and/or the failure of tumour markers to normalise by the end of three cycles BOP/BEP. Such patients received additional treatment at the discretion of the investigator. Treatment failure was defined as a plateau in the decline of tumour markers (at a level >20 IU l^−1^) on 3 successive weekly samples, or evidence of progressive disease (a rise in markers on three successive samples over a 4-week period or development of new metastatic lesions).

Treatment-related toxicity was monitored weekly through the regimen and at regular intervals after completion. Pulmonary toxicity was assessed by review of patient's symptoms and clinical examination. Pulmonary function tests and CT scans were used to further investigate new symptoms or signs. Symptoms of neuropathy were recorded and graded at each visit with clinical assessment if required. Audiometry was performed at baseline and repeated in the event of patients complaining of tinnitus or hearing loss.

### Statistical method

The study was designed as a pilot to determine the feasibility of this regimen in terms of the degree of grade 3/4 toxicity. The primary end point chosen was the percentage of patients developing grade III/IV mucositis during their chemotherapy, with <5% deemed as acceptable, >15% too toxic and in-between uncertain. It was calculated that at least 40 evaluable patients would be required to give a 90% chance of detecting an increase in the rate of grade 3/4 mucositis from 5 to 15%.

The duration of progression-free survival (PFS) and overall survival (OS) were calculated from the start of chemotherapy on an intention-to-treat basis. Actuarial survival curves were calculated using the Kaplan–Meier method. Toxicity was graded using the CTC.

## RESULTS

### Patient characteristics

A total of 43 eligible patients, from four regional testicular cancer treatment centres in the UK, were entered into this study between March 1995 and October 1999. The majority of patients were entered by two centres (51 and 37%), with differences in recruitment between the centres relating to centre size and later entry into study. The characteristics of the 43 eligible patients are summarised in Table 1

**Table 1:** Patient characteristics

Patients	43
Age at diagnosis (years): median (range)	27 (17–53)
	
	**No. of patients (%)**
*Site of primary tumour*^a^	
Testis	36 (84%)
Retroperitoneum	1 (2%)
Mediastinum	2 (5%)
Abdomen	2 (5%)
Other^b^	2 (5%)
	
*Histology*	
MTT (Malignant teratoma trophoblastic)	2 (5%)
MTU (Malignant teratoma undifferentiated)	13 (30%)
MTI (Malignant teratoma intermediate)	4 (9%)
MTD (Malignant teratoma differentiated)	3 (7%)
Other^c^	13 (30%)
Unknown^d^	8 (19%)
	
(International Germ Cell Cancer Collaborative Group) *IGCCG prognostic category*	
Intermediate	24 (56%)
Poor	19 (44%)

a In several cases, the primary biopsy of a nontesticular mass provided a tissue diagnosis, while an ultrasound of the testis revealed primary tumour. These have been included as testicular primary.

b Two patients in whom neck nodes were positive for tumour but site of primary teratoma not determined.

c Mixed seminoma/teratoma or undifferentiated tumours with elevated tumour markers.

d Histology not obtainable but elevated tumour markers diagnostic.

.

In all, 37 (86%) patients received all three cycles of BOP and BEP as planned, with 243 cycles of chemotherapy completed within the total cohort of patients. The reasons for not completing BOP/BEP chemotherapy included: psychological problems; ototoxicity; renal toxicity; pulmonary toxicity; early surgical resection of disease; and disease progression. Alternative chemotherapy was administered in all but one patient who stopped trial treatment early.

### Maintenance of dose intensity

Despite the dose intense nature of the BOP/BEP regimen, only 11 (5%) of 243 cycles of chemotherapy were administered outwith 2 days of the intended start date. Of these delays, only five can be directly ascribed to treatment, with late recovery of haematological toxicity responsible for the majority of these. Of the patients, 34 (79%) received all chemotherapy on the dates determined by protocol.

**Figure 2:**
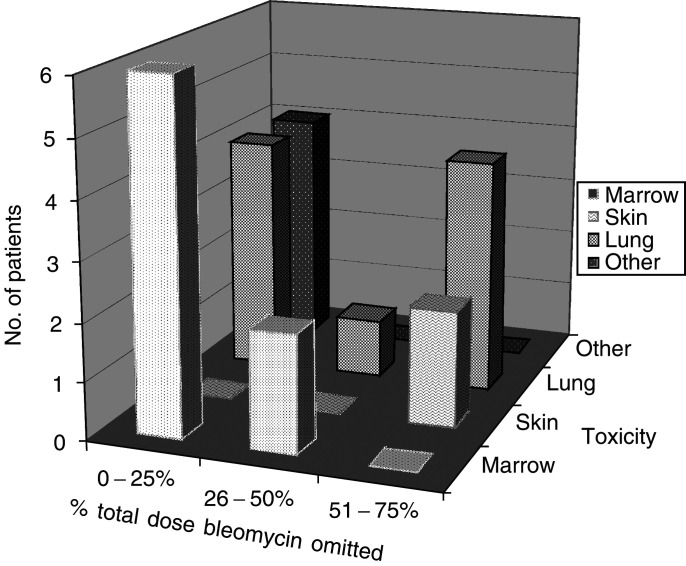
Reduction in bleomycin dose intensity due to toxicity. Percentage of dose of bleomycin omitted due to toxicity in patients receiving BOP/BEP chemotherapy.

During the course of their treatment 24 patients (56%) required a reduction in the dose of at least one of the cytotoxic agents in the BOP/BEP regimen. Most commonly, this was due to bleomycin toxicity, seen in 23 patients. Figure 2

shows the common forms of bleomycin toxicity observed and the percentage of the total planned dose omitted due to toxicity. Two patients required reductions in the dose of cisplatin in view of a fall in creatinine clearance.

### Response to treatment

In all, 25 patients (58%) achieved a complete response to BOP/BEP chemotherapy, 14 of which were initially intermediate prognosis patients and 11 were of poor prognosis. Five patients (four poor and one intermediate prognosis) had an incomplete response to chemotherapy. In four cases this was due to the presence of viable tumour in resected residual disease. Of the three with complete resection of residual tumour, one patient is currently alive and disease free, one died of surgical complications and one of disease recurrence without further chemotherapy. One patient had an incomplete resection of viable tumour, received salvage chemotherapy with high-dose carboplatin/etoposide/ifosfamide, but subsequently died from his disease. Markers failed to normalise, without obvious residual disease, in the fifth case and the patient subsequently died of relapsed disease without having further chemotherapy.

There was one treatment failure in an intermediate prognosis patient in whom markers initially fell but then started to rise again during chemotherapy.

In all, 12 patients were nonevaluable for response in that, although their markers had returned to normal with chemotherapy, there was a residual mass observed at CT scanning that was not amenable to surgical evaluation. Of these patients, five have relapsed and required further treatment while five remain alive and progression free. One patient died of treatment-related toxicity but with no evidence of active disease.

In all, 10 patients have progressed after completion of chemotherapy, all of whom have received salvage chemotherapy. Currently, four of these patients are alive and free of disease.

### Survival

**Figure 3:**
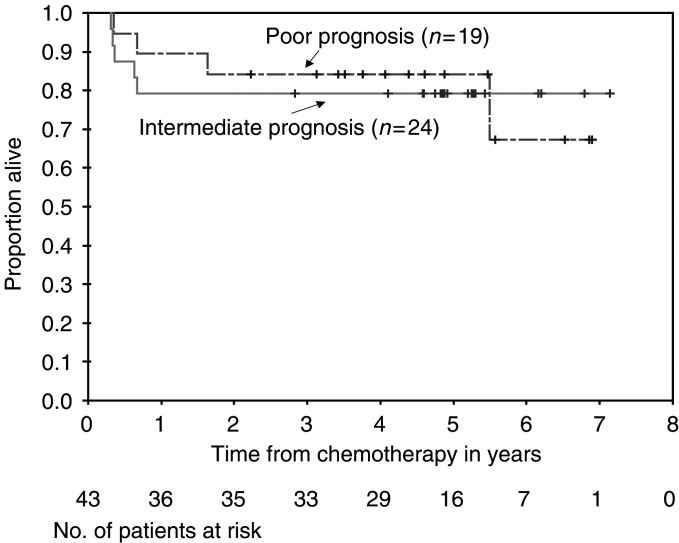
Overall survival. Percent OS of intermediate and poor prognosis patients by Kaplan–Meier curve. Number of patients available for assessment at each time point is shown.

As of June 2003, the median follow-up period for the 34 living patients is 4.9 years (range 2.2–7.1 years). The estimated proportion alive at 3 years is 81% (95% CI: 66–91%). For the intermediate prognosis group, the figure is 79% (95% CI: 57–91%), whereas for the poor prognosis group it is 84% (95% CI: 59–95%). The actuarial OS for intermediate and poor performance patients is shown in Figure 3

**Figure 4:**
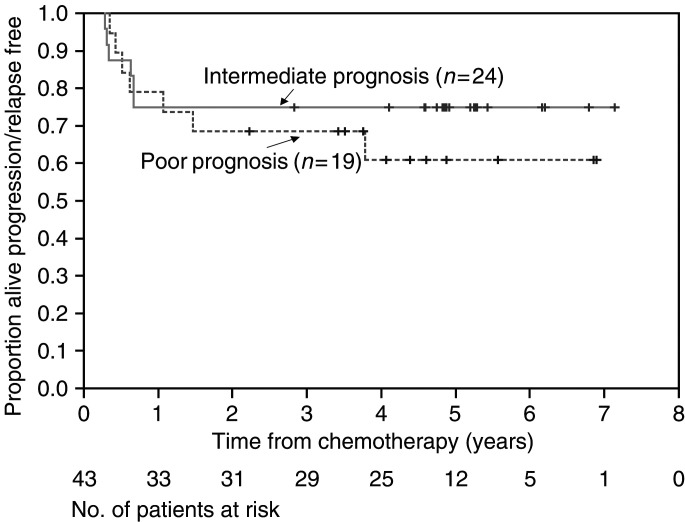
Percent PFS of intermediate and poor prognosis patients by Kaplan–Meier curve. Number of patients available for assessment at each time point is shown.

. Relapse-free survival (RFS) for all patients at 3 years was estimated at 72% (95% CI: 56–83%). Data for intermediate and poor prognosis groups are seen in Figure 4

.

Of the 42 patients evaluable for survival, nine have died to date; six from malignant disease, one from toxicity directly related to BOP/BEP chemotherapy (pulmonary fibrosis), two from complications arising after salvage therapy (renal failure during neutropenic sepsis; abdominal haemorrhage after surgery).

### Toxicity

The major toxicity was haematologic with CTC grade 3/4 neutropenia experienced by 39 patients (90%) at some time during the course of their chemotherapy (Table 2

**Table 2:** Haematological toxicity

	**Number of patients (%)**	
	**CTC grade III**	**CTC grade IV**
Granulocytopenia	4 (10%)	35 (83%)
Thrombocytopenia	8 (19%)	6 (14%)
Anaemia	10 (24%)	3 (7%)

Maximum grade of haematological toxicity experienced by patients receiving bleomycin, vincristine, cisplatin (BOP)/bleomycin, etoposide, cisplatin (BEP). CTC=common toxicity criteria.

). Anaemia and thrombocytopenia (CTC grade 3/4) occurred in 30 and 33% of patients, respectively.

In all, 13 patients required hospitalisation on 18 occasions for pyrexia associated with neutropenia. Five patients received haemopoietic growth factors (G-CSF) on a total of 10 occasions due to prolonged neutropenia or neutropenic sepsis. No septic deaths were recorded on trial chemotherapy. All other episodes resolved with appropriate antibiotic and supportive treatment.

In general, nonhaematological toxicity was mild and recovered quickly on completion of chemotherapy. The incidence of mucositis, which was the primary end point of the trial, was 7% grade 3 and no grade 4. Grade 4 biochemical toxicity (reduced Na^+^, K^+^, Mg^2+^) and grade 3 nausea, pulmonary and cutaneous toxicity was observed in a minority of patients. Two patients experienced a fall in creatinine clearance sufficient to omit cisplatin, which was subsequently reintroduced at 75% of full dose. Cisplatin-induced neurotoxicity was expected with this regimen, and Table 3

**Table 3:** Neurotoxicity of the BOP/BEP regimen

	**No. and % of patients with toxicity CTC grade 2 or >**	**No. and % of patients with toxicity lasting >1 year**
Sensory neuropathy	3 (7%)	3 (7%)
Motor neuropathy	1 (2%)	0
Ototoxicity	13 (30%)	3 (7%)

Frequency of clinically significant or persistent neurotoxicity arising with bleomycin, vincristine, cisplatin (BOP)/bleomycin, etoposide, cisplatin (BEP). CTC=common toxicity criteria.

shows the incidence of toxicity greater than CTC grade 2.

In 14 patients, significant toxicity persisted for 6 months or longer after treatment ended. This was predominantly neurosensory in the form of ototoxicity (tinnitus; hearing loss) or peripheral neuropathy (Table 3). Two patients described peripheral vasospasm on exposure to cold and two had radiological and/or physiological evidence of pulmonary fibrosis (bleomycin lung). Nine patients (none of whom received salvage chemotherapy) have indicated symptoms of residual neurosensory toxicity at review 2–4 years postchemotherapy.

As the risks of infertility (through oligospermia) were considered significant with the doses of cisplatin used in this regimen, sperm storage was offered to all patients prechemotherapy if practicable. Although the determination of fertility postchemotherapy was not routinely undertaken, at least one patient had severe oligospermia more than 2 years after treatment. Additional problems with posttreatment fertility were also encountered in the form of retrograde ejaculation after retroperitoneal lymph node dissection.

## DISCUSSION

Despite the development of strategies that successfully treat the majority of patients with advanced or metastatic germ-cell tumours (MGCT), there remains a small but significant group destined to die of their disease. The IGCCCG staging system for MGCT (IGCCCG, 1997) identifies intermediate and poor prognosis patients with 5-year progression-free survival rates of 75% and up to 53%, respectively. Although salvage chemotherapy can cure a proportion of patients after first relapse, the development of more effective first-line chemotherapy regimens for patients in poor prognostic groups remains one of the main aims of research in this field.

The current standard of care for intermediate and poor prognosis nonseminomatous MGCT is treatment with four cycles of a combination of BEP (Bosl *et al*, 1998). Attempts to improve upon this regimen, in terms of response and OS, have utilised a number of approaches, one of which has been to increase the dose intensity of treatment regimens containing drugs with established single-agent activity in germ-cell tumours. Two different forms of dose intensive regimens have been developed. (a) Induction therapy, in which several cycles of a dose intensive, relatively nonmyelotoxic, induction regimen are administered prior to the more myelotoxic drug combination. (b) Alternating therapy, using an intensive alternating schedule of myelotoxic and nonmyelotoxic drug combinations.

The original BOP/BEP regimen (Horwich *et al*, 1989) used BOP weekly as an induction regimen over a 4-week period before changing to one cycle of 5-day BEP followed by two cycles of EP (bleomycin being omitted). Bleomycin, vincristine, cisplatin given on weeks 1 and 3 was similar to that given in the current study, although bleomycin was 15 000 U. Bleomycin, vincristine, cisplatin on weeks 2 and 4 used bolus cisplatin at 40 mg m^−2^ and a 5-day continuous infusion of 75 000 U bleomycin. The total duration of chemotherapy was 13 weeks. Patients in this study were ‘high risk’ as determined by the Royal Marsden Hospital staging system. In all, 74% of evaluable patients displayed a complete response to this chemotherapy and at a median of 2 years follow–up, 85% displayed no evidence of relapse. A further adaptation of this induction-type regimen has also been studied in the phase II setting. C-BOP-BEP (Horwich *et al*, 1997; Christian *et al*, 2003) consists of BOP weeks 1 and 3, carboplatin (AUC 3 mg ml^−1^ × min) plus reduced dose BOP weeks 2 and 4, vincristine and bleomycin weeks 5 and 6 then BEP weeks 7, 10 and 13. Data on 54 patients with IGCCCG poor prognosis MGCT treated with this regimen over an 11-year period have been published recently (Christian *et al*, 2003). The 5-year OS and RFS rates were 87.6 and 83.2%, respectively. The most frequent toxicity was myelosuppresion and the only toxic death was as a result of bleomycin-induced pulmonary fibrosis. A phase II study of the BOP/VIP-B regimen, in which ifosfamide is added to the BEP component, treated 94 patients with MRC poor prognosis features (Lewis *et al*, 1992). The overall CR rate was 70% and the 2-year PFS was 63%. However, in the only randomised study to date of an induction regimen dose intense therapy, when compared to standard BEP in 380 patients, BOP/VIP-B showed greater toxicity but no improvement in response or OS (Kaye *et al*, 1998).

A dose intense, alternating schedule regimen, POMB/ACE developed at the Charing Cross Hospital in London has been used to treat patients with nonseminomatous MGCT since 1977. In a retrospective analysis of 20 years experience with this regimen (Bower *et al*, 1997), patients were classified according to the IGCCCG system. In all, 31% were of poor prognosis and 14% of intermediate prognosis prior to chemotherapy. The 3-year OS rates for these groups were calculated to be 75 and 88%, respectively. Toxicity was comparable to that published for BEP. Other centres have also had experience with POMB/ACE (Cullen *et al*, 1988; Husband and Green, 1992), but its advantage over standard BEP has never been tested in a randomised trial. BOMP/EPI is a modification of POMB/ACE, in which POMB is alternated with a modified VIP regimen. This has been used in a number of centres in Spain and over a 10-year period 38 patients, retrospectively classified as IGCCCG poor prognosis, were treated (Germa *et al*, 1999). In these patients, the CR was 49% and with a median of 41 months follow-up estimated 2-year OS and PFS were 64 and 58%.

The current study shows that a dose intense regimen, of alternating cycles of BOP and BEP, is well tolerated and produces good response rates, PFS and OS in patients with intermediate and poor prognosis MGCT. The CR rate of 58% and overall 3-year PFS of 72% (95% CI: 56–83%) compare favourably with the results from the previously mentioned dose intense studies. The analysis of the results for intermediate and poor prognosis patients suggests that most of the improvement is observed in those with a poor prognosis (3-year PFS of 68% compared to 5-year PFS of between 41 and 53% in the IGCCCG data). With a median follow-up of almost 5 years for living patients, the predicted OS at 3 years is 81% (95% CI: 66–91%), with no significant difference between intermediate and poor prognosis groups. This is similar to the POMB/ACE survival but better than that for BOMP/EPI.

The dose intense features of the alternating BOP/BEP regimen, arising principally through the increased frequency of cisplatin administration, were maintained with the vast majority of patients receiving all chemotherapy and on the protocol-determined dates. Loss of dose intensity from reductions in drug doses occurred in 56% of patients, but mainly due to the omission of bleomycin as a consequence of toxicity. However, the significance of this is unclear as five of the six patients who received less than 50% of the intended dose of bleomycin had complete responses. All the patients who had an incomplete response to chemotherapy received less than the full intended dose of BOP/BEP (one omitted cycle of BEP and five patients with from 6 to 50% of total bleomycin dose omitted).

Do the results of this study help in determining the optimum treatment for intermediate or poor prognosis NSMGCT? It is interesting to speculate as to why the benefits of this dose intense regimen appear to be predominantly in those patients with poor prognosis disease. It may be that with more extensive disease, there are a greater proportion of tumour cells with reduced sensitivity to chemotherapy and that the increased dose intensity of BOP/BEP allows less time for these cells to re-establish themselves between cycles as compared to standard BEP.

The results of this study are similar to those seen in the other phase II studies of dose intense alternating chemotherapy regimens. In light of the recently updated results of the prospectively collected data on C-BOP/BEP (Christian *et al*, 2003), the dose intense nature of BOP/BEP given by either regimen appears, in the phase II setting, to offer significant benefits in the treatment of poor prognosis MGCT compared to historical controls. Any optimism, in terms of improved outcomes in poor prognosis disease that these studies generate is, however, tempered by the negative results of the one randomised trial of this form of therapy to date (Kaye *et al*, 1998).

Is it sufficiently important to know the place of dose-intensified chemotherapy regimens, such as BOP/BEP, in the treatment of poor prognosis MGCT to justify randomised studies? The relative rarity of this group of patients makes conducting clinical trials difficult. In addition, other novel approaches are currently undergoing evaluation. The ability of high-dose chemotherapy with autologous stem-cell rescue (ASCR) to salvage heavily pretreated patients with relapsed MGCT (Beyer *et al*, 1997) has led to the examination of this approach as a possible first-line treatment in poor prognosis patients. A number of nonrandomised studies (Motzer *et al*, 1997; Bokemeyer *et al*, 1998; Decatris *et al*, 2000) have shown the practicability and tolerability of such an approach, with OS appearing better than the corresponding figure from the IGCCCG ‘test’ data. One randomised study of first-line high-dose chemotherapy has been performed (Chevreau *et al*, 1993), and this failed to show any benefit for the high-dose approach. However, the results of this study are difficult to interpret as the high-dose arm of this study had a lower dose intensity than the standard-dose arm. A recent multivariate and matched-pair comparison of poor prognosis patients receiving first-line high-dose VIP chemotherapy and those receiving standard-dose BEP or VIP suggests a significant improvement in PFS and OS for the high-dose approach (Bokemeyer *et al*, 1999a). Hopefully, the benefits of this approach will be made a little clearer on completion of a number of randomised clinical trials such as the United States intergroup study of BEP × 4 *vs* BEP × 2 followed by high-dose chemotherapy and ASCR.

The development of new cytotoxic agents and their incorporation into combination chemotherapy protocols offers another approach to improving survival in this group of patients. Paclitaxel (Motzer *et al*, 1994; Bokemeyer *et al*, 1996) and gemcitabine (Bokemeyer *et al*, 1999b; Einhorn *et al*, 1999) are two such agents and currently promising data from initial studies of Paclitaxel added to BEP (T-BEP) (de Wit *et al*, 1999) have resulted in an EORTC randomised study in intermediate prognosis MGCT comparing this regimen to BEP (EORTC protocol 30983).

The inevitable outcome of the studies into high-dose chemotherapy and combinations involving new drugs will be to present a range of treatment options for this group of patients requiring comparison in randomised trials. Determining the true value of dose intense alternating chemotherapy regimens as compared to standard BEP at this point in time could either determine a new standard regimen for this group of patients or remove one of the potential variables from future trials. A Phase III trial comparing C-BOP/BEP to standard BEP is planned in the United Kingdom, which may, hopefully, answer this question.
